# Local authority variation in special educational needs provision at the start of primary school: observational study of preterm children born in England, 2003-2013

**DOI:** 10.23889/ijpds.v9i5.2546

**Published:** 2024-09-18

**Authors:** Kate Lewis, Vincent Nguyen, Bianca De Stavola, Lorraine Dearden

**Affiliations:** 1 University College London Great Ormond Street Institute of Child Health; 2 Social Research Unit University College London

## Objective and Approach

We explored variation in special educational needs (SEN) provision between local authorities (LAs) in England by focussing on children born preterm (24-<37 weeks gestation). We used linked individual-level state-funded hospital and school records from the ECHILD database, alongside publicly available school-level records. LA of child’s residential address (n=150) and recording of SEN provision (split into SEN support and education, health and care plans, EHCPs) were captured at the January school census in year one, when pupils were five/six years old. We fitted multi-level logistic regression models to the child-level records that included a-priori selected child- and LA-level characteristics.

## Results

Our population included 226,419 children (45.4% female ; 10.6% with a congenital anomaly) born between 2003-2013 and attending a state-funded primary school. Across LAs, the median prevalence of SEN support and EHCPs recorded in year one was 18.4% (interquartile range 16.4%-20.4%) and 4.2% (3.5%-4.8%), respectively. In the SEN support vs. no SEN provision model, there was very low correlation among children from the same LA (intraclass correlation coefficient (ICC)=0.006 )). There was higher correlation in the EHCP vs. no SEN provision model (ICC=0.032). Of the LA-level characteristics, the percentage of children eligible for free school meals had the greatest impact on the ICC values (reduction of 26.7% in the EHCP model).

## Conclusions

Less than 1% of the variation in SEN support and approximately 3% of the variation in EHCPs was explained at the LA level. We will extend these models to consider variation at the school level.

